# Valorization of Bread Waste Hydrolysates and Plant-Based Nitrogen Sources for Mycoprotein Production by *Pleurotus salmoneostramineus*

**DOI:** 10.3390/foods15101773

**Published:** 2026-05-17

**Authors:** Patchana Sawetchayanont, Natta Laohakunjit, Apiradee Uthairatanakij, Orrapun Selamassakul, Kanok Ratanakanokchai, Phenjun Mekvichitsaeng, Punchira Vongsawasdi

**Affiliations:** 1 Division of Biochemical Technology, School of Bioresources and Technology, King Mongkut’s University of Technology Thonburi, Bangkok 10150, Thailand; patchana@mondenissin.co.th (P.S.); kanokrat02@gmail.com (K.R.); 2 Division of Postharvest Technology, School of Bioresources and Technology, King Mongkut’s University of Technology Thonburi, Bangkok 10150, Thailand; apiradee.uth@kmutt.ac.th; 3 Pilot Plant Development and Training Institute, King Mongkut’s University of Technology Thonburi, Bangkok 10150, Thailand; orrapun.sel@kmutt.ac.th (O.S.); phenjun.mek@kmutt.ac.th (P.M.); 4 Department of Microbiology, Faculty of Science, King Mongkut’s University of Technology Thonburi, Bangkok 10140, Thailand; punchira.von@kmutt.ac.th

**Keywords:** bread waste, mung bean protein, enzymatic hydrolysis, submerged fermentation, sustainability

## Abstract

Mycoprotein production by *Pleurotus salmoneostramineus* was evaluated using the bread industry by-products wheat bran hydrolysate (WBH) and stale bread hydrolysate (SBH) as carbon sources, with mung bean protein hydrolysate (MBH) as a nitrogen source, within a circular bioeconomy framework. Enzymatic hydrolysis effectively converted these food industry waste streams into fermentable substrates with complementary nutritional profiles: SBH provided the highest total sugar content (57.53 g/L), while MBH contributed the highest total nitrogen (3.58 g/L) and essential amino acid content (215.05 mg/100 mL). Of 11 WBH:SBH ratio formulations evaluated under static cultivation, WB4 (WBH:SBH 70:30; C/N 27.32:1) was identified as the optimal carbon source formulation, producing the highest biomass (3.46 g/L) and protein content (23.19 g/100 g) after 14 days. Subsequent nitrogen source optimization under dynamic cultivation (200 rpm, 5 days) showed that MBH supplementation at 5 g/L produced the highest biomass (16.59 g/L), protein content (66.71 g/100 g), and absolute protein production (11.07 g/L). The amino acid profile of mycoprotein produced under optimized conditions met or exceeded the FAO/WHO-recommended essential amino acid requirements for older children, adolescents, and adults; the essential amino acid content (354.72 mg/g protein) was comparable to soy protein isolate and exceeded that of wheat gluten. Mycelial morphology shifted from filamentous networks under static conditions to fragmented clump structures under dynamic cultivation with MBH supplementation. These findings indicate the feasibility of producing nutritionally complete mycoprotein from food industry waste streams, with potential applications in plant-based food formulations.

## 1. Introduction

An increasing demand for sustainable protein sources has intensified interest in alternative food ingredients with well-defined nutritional compositions. Edible mushrooms are recognized for their high-quality protein, essential amino acids, dietary fiber, and bioactive compounds, such as β-glucans and phenolics. Furthermore, their production requires fewer natural resources and generates lower greenhouse gas emissions than conventional livestock [1,2].

Mycoprotein, a fungal-derived protein produced through fermentation, represents an alternative protein source with established applications in food systems. The most widely studied example is derived from *Fusarium venenatum* using submerged fermentation [3,4]. However, other edible fungi, particularly species within the genus *Pleurotus*, have received increasing attention due to their rapid growth and favorable nutritional profiles [5,6].

Conventional production of *Pleurotus* spp. via solid-state cultivation is limited by long cultivation periods (30–48 days) and variability in environmental conditions, which affect compositional consistency. Submerged fermentation offers improved control of cultivation parameters and may enhance biomass composition reproducibility, making it more suitable for producing mycoprotein with defined nutritional characteristics [7,8]. *Pleurotus salmoneostramineus* (pink oyster mushroom; synonym, *P. djamor*) is of particular interest due to its reported protein (11.3–43.1%) and dietary fiber (7.3–12.2%) content and low fat levels (0.1–4.6%), as well as its micronutrient composition [9,10]. In addition, its natural chromoprotein-associated pigmentation may influence the physicochemical properties of its derived products [11,12]. Despite these characteristics, compositional data for mycoprotein derived from *P. salmoneostramineus* under submerged fermentation remain limited compared with other *Pleurotus* species, such as *P. ostreatus* and *P. eryngii* [5].

Agro-industrial by-products represent potential substrates for fermentation and may influence the nutritional composition of the resulting biomass. Bread waste and wheat bran are rich in carbohydrates and contain amino acids including glutamic acid, proline, leucine, and arginine [13]. Meanwhile, mung bean-derived materials provide complementary nitrogen sources, particularly lysine, the content of which is often limited in cereal-based substrates [14,15]. However, the use of solid substrates in fermentation systems complicates the determination of biomass composition due to the incorporation of residual substrate into the mycelial matrix [16]. When enzymatically hydrolyzed, mung bean protein isolate (MBPI) yields peptides and free amino acids that are readily assimilated during microbial growth [15,17]. Thus, enzymatic pre-hydrolysis provides a strategy for generating soluble substrates, facilitating more accurate assessment of biomass yield and composition.

Currently, there is a lack of information on the compositional characteristics of mycoprotein produced by *P. salmoneostramineus* when grown on combined hydrolysates derived from bread industry by-products and mung bean protein under submerged fermentation conditions. The objective of this study was therefore to investigate the effects of substrate composition and cultivation conditions on biomass production and to characterize the resulting mycoprotein in terms of protein content, amino acid profile, and microstructure.

## 2. Materials and Methods

### 2.1. Microorganism

*P. salmoneostramineus* was obtained from the Thailand Mushroom Collection Center (TMCC), Biotechnology Research and Development Office. Department of Agriculture, Chatuchak, Bangkok 10900, Thailand. The strain was maintained on potato dextrose agar (PDA) slants at 4 °C for long-term storage.

For inoculum preparation, mycelium was transferred from stock cultures onto PDA plates and incubated at 28 ± 2 °C for 10 days until sufficient growth was observed.

For static cultivation, three agar plugs (10 mm diameter) from actively growing cultures were aseptically transferred. For dynamic cultivation, three agar plugs were inoculated into 250 mL Erlenmeyer flasks containing 100 mL of culture broth and incubated at 150 rpm and 28 ± 2 °C for 5 days.

The resulting culture was homogenized for 2 min under sterile conditions, mixed with sterile glycerol (10%, *v*/*v*), and stored at −80 °C for subsequent use [18].

### 2.2. Chemicals and Reagents

Celluclast 1.5 L (≥700 U/g), Fungamyl 4000 G (4000 FAU-F/g), and Neutrase 0.8 L (≥0.8 AU/g) were obtained from Novonesis under distributor Brenntag Ingredients (Thailand) Public Company Limited 1168/98-100 Lumpini Tower 33rd Floor, Rama IV Road, Thungmahamek, Sathorn 10120, Bangkok, Thailand. Stem bromelain (≥3 U/mg protein), MBPI, and soy peptone were obtained from Longevity Thailand 50 Ramindra 14, Bangkok, Thailand. L-Methionine (MET) and glycine (GLY) were purchased from AJITRADE (THAILAND) CO., LTD. Si-Ayutthaya Building, 13th Floor, 487/1 Si Ayutthaya Road, Ratchathewi, Bangkok 10400, Thailand. Potato dextrose agar (PDA), potato dextrose broth (PDB), malt extract, and yeast extract were obtained from HiMedia Laboratories (India) under distributor M&P IMPEX LTD PART 2/8 Chao Khun Thahan Road, Lam Pla Thio, Lat Krabang, Bangkok 10520, Thailand. Glucose, K_2_HPO_4_, and MgSO_4_·7H_2_O (analytical grade) were supplied by VALUE INDUSTRIAL PRODUCTS Co., Ltd. (Thailand). 229/17 Saphansoong, Bangkok 10240, Thailand.

### 2.3. Preparation of Hydrolysates

#### 2.3.1. Enzymatic Wheat Bran Hydrolysate (WBH)

Wheat bran (WB) was obtained from Monde Nissin Corporation Felix Reyes St., Barangay Balibago, Sta. Rosa City, Laguna, 4026 Philippines, with a composition of 17.94% protein, 3.22% fat, 3.80% ash, 61.01% carbohydrate, and 14.02% moisture, as determined in duplicate according to AOAC method [19]. Prior to enzymatic hydrolysis, WB was autoclaved at 121 °C for 15 min and stored at 6 ± 2 °C. The sterilized WB was then mixed with distilled water at a ratio of 1:20 (*w*/*v*) to form a suspension. Celluclast (0.4%, *w*/*w*) and Neutrase (0.4%, *w*/*w*) were added, and the pH was adjusted to 5.0 using 1 M NaOH. Enzymatic hydrolysis was performed in a 45 L shaking water bath (Memmert GmbH + Co. KG, Schwabach, Germany) at 75 °C for 6 h. The reaction was terminated by heating at 95 °C for 15 min. The hydrolysate was subsequently centrifuged at 2800× *g* for 20 min at 25 °C, and the supernatant was collected as substrate for analysis of total nitrogen, total sugar, and total amino acid content.

#### 2.3.2. Enzymatic Stale Bread Hydrolysate (SBH)

Stale bread (SB), defined as bread approaching its expiration date (1 day prior to expiry), was obtained from a food company via Makro Food Service, Prachautid Branch, Bangkok, Thailand; the sample composition included 9.29% protein, 1.58% fat, 4.21% ash, 49.70% carbohydrate, and 35.22% moisture, as determined in duplicate according to AOAC method [19]. Microbiological analysis was conducted using 3M Petrifilm plates (3M Food Safety, St. Paul, MN, USA). Total plate count (TPC), coliforms, and *Escherichia coli* were determined according to AOAC Official Methods 990.12 and 991.14, [20,21] respectively. Yeast and mold counts were analyzed following AOAC Official Method 997.02 [22] The results showed that all microbial counts were within acceptable limits (TPC < 1 × 10^4^ CFU/g; coliforms and *E. coli* < 10 CFU/g; yeast and mold < 100 CFU/g). Following a modified version of the methodology from Benabda et al. [23], SB was diced, dried at 135 °C for 15 min, ground into powder, autoclaved at 121 °C for 15 min, and stored at 6 ± 2 °C. The sterilized SB powder was then mixed with distilled water at a ratio of 1:10 (*w*/*v*) and heated at 60 °C for 15 min to promote starch gelatinization. Fungamyl (0.4%, *w*/*w*) and Neutrase (0.2%, *w*/*w*) were added, and the pH was adjusted to 6.0 using 1 M NaOH. The mixture was incubated in a 45 L shaking water bath (Memmert GmbH + Co. KG, Schwabach, Germany) at 60 °C for 90 min. The reaction was terminated by heating at 95 °C for 15 min. After centrifugation (2800× *g*, 20 min, 25 °C), the supernatant was collected as substrate for analysis of total nitrogen, total sugar, and total amino acid content.

#### 2.3.3. Enzymatic Mung Bean Hydrolysate (MBH)

MBPI was used as a nitrogen source. It consisted of 83.70% protein, 0.24% fat, 8.48% ash, 7.58% carbohydrate, and 0.06% moisture, as determined in duplicate according to AOAC method [19]. Following a method modified from Sonklin et al. [15], MBPI was dispersed in distilled water at a ratio of 1:5 (*w*/*v*). The pH was adjusted to 6.0, and stem bromelain (15%, *w*/*w*) was added. The mixture was incubated in a 45 L shaking water bath (Memmert GmbH + Co. KG, Schwabach, Germany) at 50 °C for 6 h. The reaction was terminated by heating at 95 °C for 15 min. After centrifugation (2800× *g*, 20 min, 25 °C), the supernatant was collected as substrate for analysis of total nitrogen, total sugar, and total amino acid content.

### 2.4. Characterization of Raw Materials

#### 2.4.1. Chemical Composition of WB, SB, and MBPI

The chemical compositions (protein, carbohydrate, fat, ash, and moisture) of WB, SB, and MBPI were determined according to AOAC method [19]. Protein was quantified using the Kjeldahl method (N × 6.25), fat by Soxhlet extraction, moisture by oven drying at 105 °C to constant weight, and ash by furnace combustion at 550 °C to constant weight. Carbohydrate content (%) was calculated from the difference: 100% − (moisture + protein + ash + fat)%.

#### 2.4.2. Total Nitrogen, Total Sugar, and Carbon-to-Nitrogen Ratio of Hydrolysates

The total nitrogen contents of wheat bran hydrolysate (WBH), stale bread hydrolysate (SBH), and mung bean hydrolysate (MBH) were determined using the Kjeldahl method [19], and total sugar was measured with the phenol–sulfuric acid method [24]. The carbon-to-nitrogen (C/N) ratio was calculated according to Equation (1):

C/N = Total sugar (g/L)/Total nitrogen (g/L)(1)

#### 2.4.3. Amino Acid Composition of WBH, SBH, and MBH

Samples were hydrolyzed with 6 N HCl at 110 °C for 24 h under a nitrogen atmosphere, followed by derivatization using the AccQ-Fluor reagent kit (Milford, MA, USA).

Amino acids were separated and quantified using high-performance liquid chromatography (Alliance 2695; Waters Corp., Milford, MA, USA) equipped with a fluorescence detector. Quantification was performed using an external standard mixture containing 15 amino acids.

### 2.5. Effect of Carbon Sources Under Static Conditions

Culture media containing different ratios of wheat bran hydrolysate (WBH) and stale bread hydrolysate (SBH) were prepared (*w*/*w*) as follows (WBH:SBH): WB1 (100:0), WB2 (90:10), WB3 (80:20), WB4 (70:30), WB5 (60:40), WB6 (50:50), WB7 (40:60), WB8 (30:70), WB9 (20:80), WB10 (10:90), and WB11 (0:100). PDB was used as a control. Each medium contained 73.02 g/L hydrolysate mixture supplemented with glucose (20 g/L), malt extract (5 g/L), yeast extract (0.5 g/L), K_2_HPO_4_ (1.18 g/L), and MgSO_4_·7H_2_O (0.5 g/L). The initial pH was adjusted to 5.5 using 1 M NaOH prior to sterilization at 121 °C for 15 min. Initial residual sugar was determined using the 3,5-dinitrosalicylic acid (DNS) method [25]. Three agar plugs (10 mm diameter; approximately 3 g) from 10-day-old cultures of *P. salmoneostramineus* were aseptically transferred into 250 mL Erlenmeyer flasks containing 100 mL medium (*n* = 3) and incubated under static conditions at 28 ± 2 °C. Aliquots (10 mL) were collected on days 7 and 14 and centrifuged at 1789× *g* for 10 min. On day 7, pH and biomass were determined. At the end of cultivation (day 14), the supernatant was analyzed for pH and residual sugar content. The harvested biomass was freeze-dried for biomass determination and protein analysis. The optimal carbon source formulation was selected for further nitrogen source experiments.

### 2.6. Effect of Nitrogen Sources Under Dynamic Conditions

The optimal carbon source formulation identified in Section 2.5 (73.02 g/L) was used as the basal medium and supplemented with glucose (20 g/L), malt extract (5 g/L), yeast extract (0.5 g/L), K_2_HPO_4_ (1.18 g/L), and MgSO_4_·7H_2_O (0.5 g/L). Four nitrogen sources (methionine (MET), glycine (GLY), soy peptone (PEP), and MBH) were evaluated at concentrations of 1, 3, and 5 g/L. The initial pH was adjusted to 5.5 using 1 M NaOH prior to sterilization at 121 °C for 15 min. For dynamic cultivation, 5% (*v*/*v*) inoculum from *P. salmoneostramineus* stock culture was transferred into 250 mL Erlenmeyer flasks containing 100 mL medium (*n* = 3) and incubated at 200 rpm and 25 ± 2 °C for 5 days. At the end of cultivation (day 5), aliquots (10 mL) were collected and centrifuged at 1789× *g* for 10 min. The supernatant was analyzed for pH and residual sugar content, and the biomass was freeze-dried for biomass and protein determination.

### 2.7. Characteristics of Biomass

#### 2.7.1. pH and Residual Sugar

The pH of the culture supernatant was measured using a pH meter (Mettler-Toledo, Greifensee, Switzerland).

Residual sugar was determined using the DNS method [25]. Briefly, 0.5 mL diluted supernatant (1:25) was mixed with 0.5 mL DNS reagent, heated at 100 °C for 5 min, cooled in an ice-water bath for 5 min, diluted with 5 mL distilled water, and measured at 540 nm using a spectrophotometer (UV-1800, Shimadzu, Japan). Residual sugar concentration was calculated from a glucose standard curve. Sugar consumption was calculated using Equation (2):

Sugar consumption (g/L) = Initial sugar (g/L) − Residual sugar (g/L)(2)

#### 2.7.2. Biomass Determination

Mycelial biomass was collected by centrifugation at 1789× *g* for 10 min, washed three times with distilled water, freeze-dried, and weighed using an analytical balance (Mettler-Toledo, Greifensee, Switzerland). Biomass concentration is expressed as dry weight (g/L).

#### 2.7.3. Protein Production

The protein content of freeze-dried biomass was determined using the Kjeldahl method (N × 6.25) and expressed as g/100 g dry weight [26]. Protein production (g/L) was calculated by multiplying biomass concentration by protein content.

### 2.8. Characterization of Mycoprotein

#### 2.8.1. Amino Acid Composition

Freeze-dried biomass obtained under optimal static and dynamic conditions (based on biomass and protein content) was analyzed as described in Section 2.4.3. Results are expressed as mg/g protein.

#### 2.8.2. Microstructure Analysis

Selected freeze-dried biomass samples were analyzed using scanning electron microscopy (SEM) (JSM-IT300, JEOL Ltd., Tokyo, Japan). Samples were mounted on aluminum stubs using double-sided carbon tape, sputter-coated with gold under vacuum, and observed at an accelerating voltage of 10 kV. Micrographs were obtained at magnifications of 20× and 1000×.

### 2.9. Statistical Analysis

The carbon source experiment (Section 2.5) was conducted using a completely randomized design (CRD) with three replicates per treatment. The nitrogen source experiment (Section 2.6) followed a 4 × 3 factorial design in CRD, with nitrogen source type (MET, GLY, PEP, and MBH) and concentration level (1, 3, and 5 g/L) as factors, with three replicates per treatment. All analyses were performed in triplicate. Data are expressed as mean ± standard deviation. Statistical analysis was performed using IBM SPSS Statistics (version 20; IBM Corp., Armonk, NY, USA; https://www.ibm.com/products/spss-statistics, accessed on 13 May 2026) Differences among means were evaluated using Duncan’s multiple range test at *p* ≤ 0.05.

## 3. Results and Discussion

### 3.1. Chemical Composition of Substrates

The proximate compositions of WB, SB, and MBPI are reported in Section 2.3.1 and Section 2.3.2. The carbohydrate content of SB (49.70%) is reflected in the greater total sugar yield of SBH 57.53 g/L following enzymatic hydrolysis compared to those of WBH (6.44 g/L) and MBH (22.89 g/L) (*p* ≤ 0.05, Table 1). During SBH preparation, starch gelatinization at 60 °C before enzyme addition disrupts the crystalline starch granule structure, increasing accessibility for enzymatic attack [23]. The subsequent addition of α-amylase catalyzes the hydrolysis of α-1,4-glycosidic linkages within gelatinized starch chains, generating maltodextrins and reducing sugars; simultaneously, the protease cleaves peptide bonds within gluten and other storage proteins, releasing soluble peptides and free amino acids [27]. The combined action of these two enzymes effectively converts the starch and protein fractions of SB into readily fermentable and assimilable nutrients.

In contrast, the lower sugar yield from WBH (6.44 g/L) reflects the more complex carbohydrate architectures in its composition [14]. Cellulase hydrolyzes β-1,4-glycosidic linkages within cellulose chains, releasing cellobiose and glucose monomers from the cellulosic fraction. The protease simultaneously targets the protein fraction of WB (17.94%), cleaving peptide bonds to release soluble nitrogen compounds, consistent with the higher observed total nitrogen content of WBH (2.58 g/L) relative to SBH (1.32 g/L; *p* ≤ 0.05, Table 1). However, despite this dual enzymatic treatment, the lower sugar yield of WBH compared with SBH may be attributed to the lignocellulosic nature of WB, which contains complex hemicellulose and lignin structures that can limit enzyme accessibility. Complete deconstruction of lignocellulosic substrates typically requires a broader enzyme system, including hemicellulases and ligninases [28].

For MBH, bromelain cleaves peptide bonds, generating a diverse mixture of peptides and free amino acids from the globulin and albumin fractions of MBPI [15,17]. This resulted in the highest total nitrogen content (3.58 g/L) among the three hydrolysates (*p* ≤ 0.05). The moderate total sugar content (22.89 g/L) of MBH likely originated from the carbohydrate fraction (7.58%) of MBPI, which is partially solubilized during the hydrolysis process.

Enzymatic hydrolysis increased the total amino acid content of all substrates relative to their unhydrolyzed equivalents (Table S1). The total amino acid content of WBH increased from 12.98 mg/100 mg (WB) to 449.47 mg/100 mL, that of SBH increased from 12.54 mg/100 mg (SB) to 96.64 mg/100 mL, and that of MBH from 17.46 mg/100 mg (MBPI) to 588.06 mg/100 mL. This demonstrates that enzymatic treatment effectively cleaved protein structures and released bound amino acids into soluble fractions available for fungal assimilation. WBH content was dominated by glutamic acid (136.37 mg/100 mL) and proline (47.88 mg/100 mL), reflecting the glutamine- and proline-rich composition of wheat gluten proteins [13]. Meanwhile, MBH exhibited the highest total contents of essential amino acids (EAAs; 215.05 mg/100 mL) and hydrophobic amino acids (HAA; 205.61 mg/100 mL) among the three hydrolysates; this is consistent with the well-balanced amino acid composition of mung bean protein [15].

The profiles of the three hydrolysates, including SBH as the primary carbon source, WBH as a supplementary carbon and nitrogen contributor enriched in glutamic acid, and MBH as the principal nitrogen source with a balanced EAA profile, collectively provide a nutrient-rich medium suitable as a carbon and nitrogen source for submerged fermentation.

### 3.2. Effect of Carbon Sources Under Static Conditions

At day 7, pH values across all formulations declined relative to the initial pH of 5.5 to values ranging from 4.36 to 5.92 (Table S2). This possibly reflects the accumulation of metabolic organic acids during active substrate utilization, as previously reported in submerged fungal cultivation studies [29]. Formulations with higher SBH proportions (WB5–WB11) showed greater acidification, with pH values ranging from 4.36 to 4.90, consistent with more rapid fermentation of the higher available sugar content. By day 14, pH values recovered toward near-neutral levels, ranging from 6.40 to 6.74 across all formulations (Table 2). This recovery may be due to the depletion of fermentable sugars, reducing acid production, combined with the possible release of ammonium ions from amino acid and protein catabolism during the later cultivation stage [8,29]. The near-neutral pH at day 14 indicates that the cultures transitioned from active sugar fermentation toward protein and amino acid catabolism as the primary metabolic activity.

Sugar consumption at day 14 was highest in WB3 (23.77 g/L), followed by WB4 (22.87 g/L) and WB2 (22.86 g/L); there were no significant differences among these three formulations (*p* > 0.05, Table 2). Sugar consumption decreased in WB6–WB11, ranging from 19.39 to 20.50 g/L. This pattern is consistent with carbon catabolite suppression, during which high glucose concentrations suppress the uptake of alternative carbon sources, thereby limiting the overall efficiency of substrate utilization [30].

Mycelium biomass at day 7 was highest in WB4 (2.30 g/L; Table S2), significantly exceeding all other formulations (*p* ≤ 0.05). The increase continued to 3.46 g/L at day 14 (Table 2), indicating that the nutritional composition of WB4 supported sustained mycelial development throughout the cultivation period. This biomass accumulation from day 7 to day 14 corresponded with the highest residual sugar consumption in WB4 (22.87 g/L), suggesting that available carbon was efficiently directed toward mycelial growth. The sustained biomass production in WB4 may be due to the C/N ratio (27.32:1) and amino acid composition of the medium. As shown in Table S3, WB4 had a total amino acid content of 561.86 mg/100 mL, dominated by glutamic acid (170.46 mg/100 mL) and proline (59.85 mg/100 mL); these acids are known to serve as key nitrogen donors in fungal transamination reactions and to support anabolic activity during mycelial growth. The comparatively lower biomass observed in formulations WB1–WB3, despite adequate nitrogen availability from WBH, may be partly attributed to the higher fat content of WB as a raw material (3.22% dry matter) relative to SB (1.58% dry matter). Lipids present in cereal-based substrates are known to form starch–lipid complexes that resist enzymatic hydrolysis, reducing the accessibility of starch to amylolytic enzymes and limiting the release of fermentable sugars [31,32]. This is consistent with the low total sugar content observed in WBH (6.44 g/L) relative to SBH (57.53 g/L, Table 1). The lower fat content of WB4 (70:30) may have improved enzymatic hydrolysis efficiency, contributing to better biomass production, alongside the favorable C/N ratio (27.32:1). In contrast, formulations with higher SBH proportions (WB6–WB11) produced lower biomass, ranging from 2.12 to 2.45 g/L at day 14. This was despite higher C/N ratios and sugar availability, indicating that reduced nitrogen from lower WBH proportions restricted fungal growth. Furthermore, as *P. salmoneostramineus* is an aerobic basidiomycete, static cultivation likely imposed an additional constraint, limiting oxygen transfer [33]. Oxygen supply under static conditions relies primarily on passive diffusion, potentially limiting aerobic metabolism and overall biomass formation.

Protein content at day 14 was highest in WB4 (23.19 g/100 g), significantly exceeding WB5–WB11 (*p* ≤ 0.05, Table 2); however, WB0 (21.79 g/100 g) and WB1 (22.30 g/100 g) were not significantly different from WB4 (*p* > 0.05). Protein content decreased progressively with increasing SBH proportion, ranging from 12.11 in WB11 to 14.80 g/100 g in WB7, consistent with reduced nitrogen availability as the proportion of WBH decreased. Although all formulations were supplemented with yeast extract (0.5 g/L) as a basal nitrogen source, the lower nitrogen input in the WB5–WB11 compositions is insufficient to fully support protein biosynthesis in the relatively carbon-rich conditions provided by the lower WBH:SBH ratios. The combination of the highest biomass (3.46 g/L) and protein content (23.19 g/100 g) in WB4 resulted in the highest absolute protein production (0.80 g/L). Nevertheless, the protein content achieved under carbon-source optimization alone remained low relative to the potential of *P. salmoneostramineus* under nitrogen-sufficient conditions. This indicates that nitrogen supplementation is the primary limiting factor for further enhancement of mycoprotein yield and protein accumulation. Based on these results, WB4 (70:30 WBH:SBH, C/N = 27.32:1) was identified as the optimal carbon source formulation and was selected for subsequent nitrogen source optimization under dynamic conditions.

### 3.3. Effect of Nitrogen Sources Under Dynamic Conditions

Nitrogen source type and concentration significantly influenced final pH values (*p* ≤ 0.05, Table 3). All tested nitrogen sources, including single amino acids (MET and GLY) and complex hydrolysates (PEP and MBH), were thoroughly dissolved in the medium prior to sterilization, ensuring their bioavailability for mycelial uptake. All nitrogen-supplemented treatments exhibited lower pH values (4.45–5.38) than the control (6.52). This decrease may be due to amino acid catabolism, in which deamination reactions generate organic acid intermediates (e.g., pyruvate and oxaloacetate) that accumulate and reduce the pH [29]. In addition, amino acids contain both carboxyl (-COOH) and amino (-NH_2_) groups, the ionization of which contributes to proton release under active metabolism, further promoting acidification [34]. GLY supplementation resulted in the mildest acidification (pH 5.25–5.37). This is consistent with its relatively simple metabolic role in one-carbon metabolism and purine biosynthesis, which generates fewer acidic by-products. In contrast, MET showed a variable pH response (4.67–5.38), possibly due to the catabolism of its sulfur-containing side chain, which can produce acidic intermediates. Complex nitrogen sources produced more pronounced and consistent acidification, with final pH values ranging from 4.52 to 4.67 for PEP and 4.45 to 4.71 for MBH. This likely reflects the broader amino acid spectrum they provide, which activates multiple metabolic pathways and increases the accumulation of organic acid intermediates [29]. Nevertheless, all pH values remained within the range suitable for *Pleurotus* growth (pH 4.2–7.5), with optimal growth typically observed between pH 5.0 and 6.5 [34].

Sugar consumption was significantly affected by the type and concentration of the nitrogen source (*p* ≤ 0.05, Table 3). MBH supplementation resulted in the highest sugar consumption, increasing dose-dependently (25.57, 30.36, and 35.43 g/L at 1, 3, and 5 g/L, respectively), significantly exceeding the control (22.87 g/L). This indicates that MBH effectively supported carbon utilization through its balanced amino acid composition. In contrast, MET resulted in the lowest sugar consumption (17.50, 21.31, and 20.46 g/L at 1, 3, and 5 g/L), possibly reflecting limited metabolic integration when only a single, sulfur-containing amino acid is supplied [35]. GLY and PEP showed intermediate sugar consumption (18.44–19.68 g/L) without a clear dose-dependent trend. Biomass production was significantly influenced by nitrogen source type and concentration (*p* ≤ 0.05, Table 3). MBH supplementation produced the highest biomass, increasing in a dose-dependent manner (10.22, 11.30, and 16.59 g/L at 1, 3, and 5 g/L, respectively), with WB4 + MBH5 (16.59 g/L) significantly exceeding all other treatments (*p* ≤ 0.05). PEP supplementation also increased biomass (7.65–11.51 g/L), with a positive dose–response dependency, while GLY resulted in moderate biomass (5.27–9.68 g/L). In contrast, MET produced biomass values below those of the control (1.56–2.38 g/L), suggesting a potential inhibitory effect when used as the sole nitrogen source. Furthermore, while inorganic nitrogen sources (e.g., ammonium sulfate or nitrates) are commonly used in synthetic media, in studies on *P. citrinopileatus* by Wu et al. [36], yeast peptone yielded the highest biomass (45.25%), whereas inorganic nitrogen sources such as NaNO_3_ and (NH_4_)_2_SO_4_ resulted in substantially lower biomass (15.95% and 5.17%, respectively). This difference can be attributed to the metabolic requirement for nitrogen assimilation. Inorganic nitrogen must first be converted into amino acids through energy-dependent pathways, whereas organic nitrogen sources provide readily assimilable amino acids and peptides, facilitating faster protein synthesis and cell growth. Previous studies using glucose-based media reported biomass production of approximately 16.75 g/L for *P. ostreatus*, although extended cultivation periods of up to 26 days were required [37]; inorganic nitrogen sources were excluded in this study, as they typically support significantly lower biomass and protein production in higher fungi compared to organic sources. The superior biomass observed with MBH supplementation may be related to enrichment of the amino acid profile (Table S3), particularly the increased content of lysine (from 18.74 to 83.97 mg/100 mL), glutamic acid (from 170.46 to 351.01 mg/100 mL), and total EAAs (from 187.52 to 538.56 mg/100 mL), which support efficient nitrogen assimilation and anabolic metabolism [38]. The substantial increase in biomass under dynamic conditions highlights the ability of agitation to improve oxygen transfer and mixing efficiency, thereby sustaining aerobic metabolism and enhancing substrate uptake. Adebayo et al. [39] reported biomass of 2.5 g/L after 14 days for *P. ostreatus* supplemented with GLY, whereas the present study achieved 16.59 g/L within 5 days under dynamic conditions with MBH. This suggests combined benefits of complex nitrogen supplementation and agitation.

Protein content was highest in WB4 + PEP5 (66.93 g/100 g) and WB4 + MBH5 (66.71 g/100 g) formulations, with no significant difference between the two (*p* > 0.05) and both significantly higher than the control (23.54 g/100 g; *p* ≤ 0.05). This improvement suggests that dynamic cultivation combined with complex nitrogen supplementation enhances protein biosynthesis, likely through improved oxygen availability and ATP generation [29]. MET supplementation resulted in moderate protein content (31.32–37.37 g/100 g) despite low biomass, suggesting preferential incorporation into cellular protein rather than biomass expansion. GLY produced the lowest protein content among the nitrogen-supplemented treatments (30.32–32.52 g/100 g), which may reflect its limited efficiency as a sole nitrogen source under submerged fermentation conditions. In contrast, glycine supplementation has been reported to enhance biomass production in other fungal species; for example, the addition of glycine (500 mg/L) increased fruiting body biomass of *Cordyceps militaris* by up to 77.22% in solid-state cultivation [40]. The inclusion of MET and GLY in this study served as a benchmark to evaluate the metabolic impact of single versus complex nitrogen sources. While complex sources are more industrially relevant, these single amino acids give a clear demonstration of how nitrogen source diversity influences anabolic efficiency in *P. salmoneostramineus*. Discrepancies may be attributed to differences in fungal species, cultivation systems, and metabolic requirements, indicating that the utilization of glycine is highly context- and species-dependent. Although PEP and MBH at 5 g/L produced comparable protein contents, the higher biomass with MBH resulted in greater protein production (11.07 g/L) than PEP (7.73 g/L). The protein content achieved under optimized conditions (66.71–66.93 g/100 g) exceeded values reported for *P. ostreatus* grown on PEP (37.9 g/100 g) and WB (34.5 g/100 g).

These results indicate that the use of bread industry by-product hydrolysates as combined carbon and nitrogen sources provides a more nutritionally balanced medium for protein accumulation than single nitrogen source supplementation. Based on these results, WB4 supplemented with MBH at 5 g/L under dynamic conditions was identified as the optimal formulation for *P. salmoneostramineus* mycoprotein production and was selected for further characterization.

### 3.4. Mycelium Characteristics

#### 3.4.1. Total Amino Acid Composition

The total amino acid content of freeze-dried mycelium increased from 109.21 mg/g protein under static WB4 cultivation to 320.11 mg/g protein under the dynamic equivalent, representing a 193% increase (Table 4). A further increase of 138% was observed with 5 g/L MBH supplementation under dynamic conditions (200 rpm and 25 ± 2 °C for 5 days), reaching 762.30 mg/g protein. This progressive enrichment reflects the combined effects of agitation and nitrogen supplementation on amino acid biosynthesis and accumulation in the mycoprotein [41,42].

Total EAA content increased from 49.53 mg/g protein under static WB4 conditions to 143.17 mg/g protein under dynamic conditions, and further to 354.72 mg/g protein with 5 g/L MBH supplementation. The proportion of EAA was highest under dynamic WB4 conditions (46.53%), suggesting that agitation alone enhanced EAA biosynthesis, likely through improved oxygen transfer and respiratory activity. The comparative analysis between the MBH substrate (Table 1) and the resulting mycelium reveals a clear transformation mechanism. The abundance of specific EAAs in MBH, such as lysine (57.69 mg/100 mL) and leucine (40.99 mg/100 mL), served as a critical metabolic input. In fungi, these exogenous amino acids can be efficiently absorbed via specialized peptide transporters (PTR), bypassing energy-intensive de novo synthesis pathways [43]. This selective uptake and bioaccumulation resulted in a significantly enriched mycelial amino acid profile, particularly in terms of lysine and leucine (50.88 and 67.41 mg/g protein, respectively), meeting the FAO requirements for human nutrition. In fungi, amino acid biosynthesis is closely linked to central carbon metabolism. Glycolysis contributes to the formation of alanine, glycine, valine, and leucine, while the tricarboxylic acid cycle generates aspartic acid, glutamic acid, and lysine [44]. Under WB4 + MBH5 conditions, the external supply of amino acids from MBH likely reduced the metabolic energy required for de novo synthesis, allowing greater carbon flux to be directed toward amino acid accumulation, resulting in the highest EAA content (354.72 mg/g protein).

Beyond the primary nutritional profile, *P. salmoneostramineus* demonstrated a robust biosynthetic capacity for specific amino acids. Notably, the high accumulation of glutamic acid (109.18 mg/g protein) contributes to the sensory umami value [45], while the high lysine content (50.88 mg/g protein) effectively complements cereal-based diets, which are typically lysine-deficient [46]. Additionally, leucine content (67.41 mg/g protein) is nutritionally relevant due to its role in stimulating muscle protein synthesis [47]. According to comparison with FAO amino acid scoring patterns, mycoprotein produced under WB4 + MBH5 dynamic conditions met or exceeded the recommended levels of all EAAs for older children, adolescents, and adults (Table 4). Lysine was markedly increased from 5.93 to 50.88 mg/g protein (758% increase), exceeding the adult requirement (48 mg/g protein). Leucine (67.41 mg/g protein), isoleucine (39.94 mg/g protein), and valine (43.03 mg/g protein) also exceeded recommended levels (30–40 mg/g protein). The combined phenylalanine and tyrosine content (66.29 mg/g protein) satisfied FAO requirements.

The EAA content obtained under dynamic conditions (354.72 mg/g protein) is comparable to that of soy protein isolate (350–450 mg/g protein), and higher than values reported for wheat gluten (250–350 mg/g protein) [48]. These data support the potential of *P. salmoneostramineus* mycoprotein as a high-quality protein ingredient. In addition, the high leucine content is nutritionally relevant due to its role in stimulating muscle protein synthesis and regulating metabolic signaling pathways [47]. Glutamic acid and aspartic acid were the predominant amino acids under dynamic WB4 + MBH5 conditions, reaching 109.18 and 94.04 mg/g protein, respectively. Compared with static WB4 cultivation, glutamic acid increased by 597% and aspartic acid by 617%, indicating efficient assimilation of exogenous amino acids from MBH (Section 3.1). Glutamic acid serves as a central nitrogen donor in fungal transamination reactions, while both glutamic and aspartic acids contribute to umami taste, potentially enhancing the sensory properties of mycoprotein-based products [49].

Total HAA content increased from 45.29 mg/g protein under static WB4 conditions to 299.51 mg/g protein under dynamic WB4 + MBH5 conditions. This increase is relevant to functional protein properties, as hydrophobic amino acids play key roles in protein folding and interfacial behavior, influencing emulsification, gelation, and foaming capacity [50]. Therefore, the mycoprotein produced under dynamic conditions may exhibit favorable techno-functional properties in terms of plant-based food applications.

#### 3.4.2. Microstructure of *P. salmoneostramineus* Mycelium

**Figure 1:**
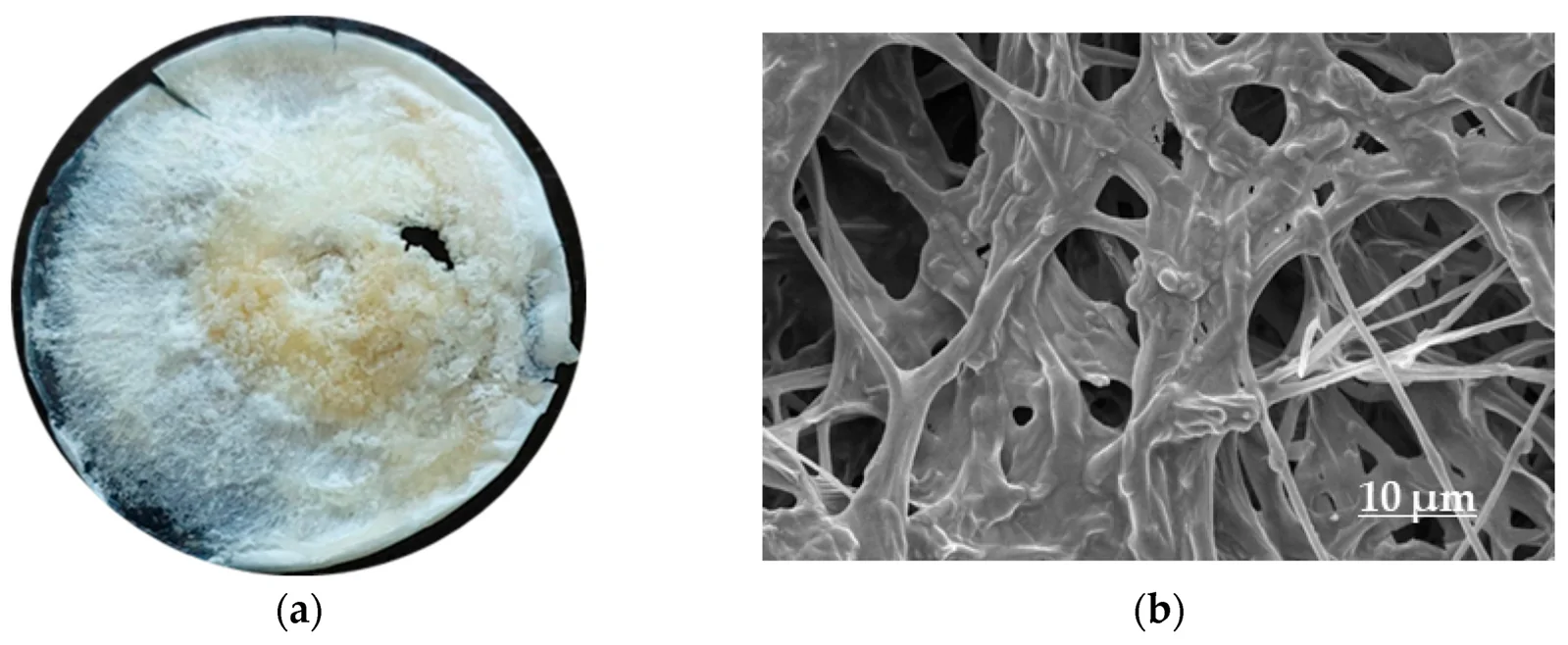
*P. salmoneostramineus* mycelium cultivated in potato dextrose broth medium under static conditions, 14 days: (**a**) photo of Petri dish 1×; (**b**) scanning electron microscopy 1000×.

**Figure 2:**
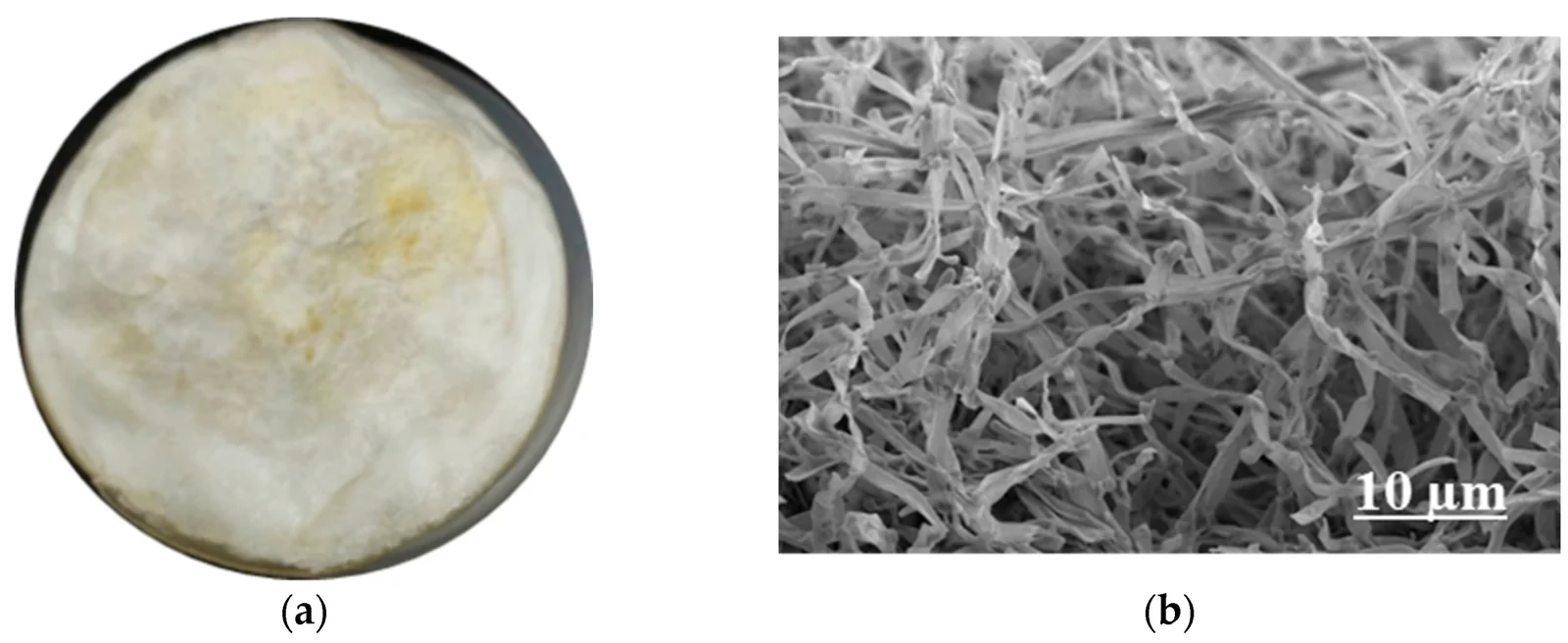
*P. salmoneostramineus* mycelium cultivated in WB4 medium under static conditions, 14 days: (**a**) photo of Petri dish 1×; (**b**) scanning electron microscopy 1000×.

**Figure 3:**
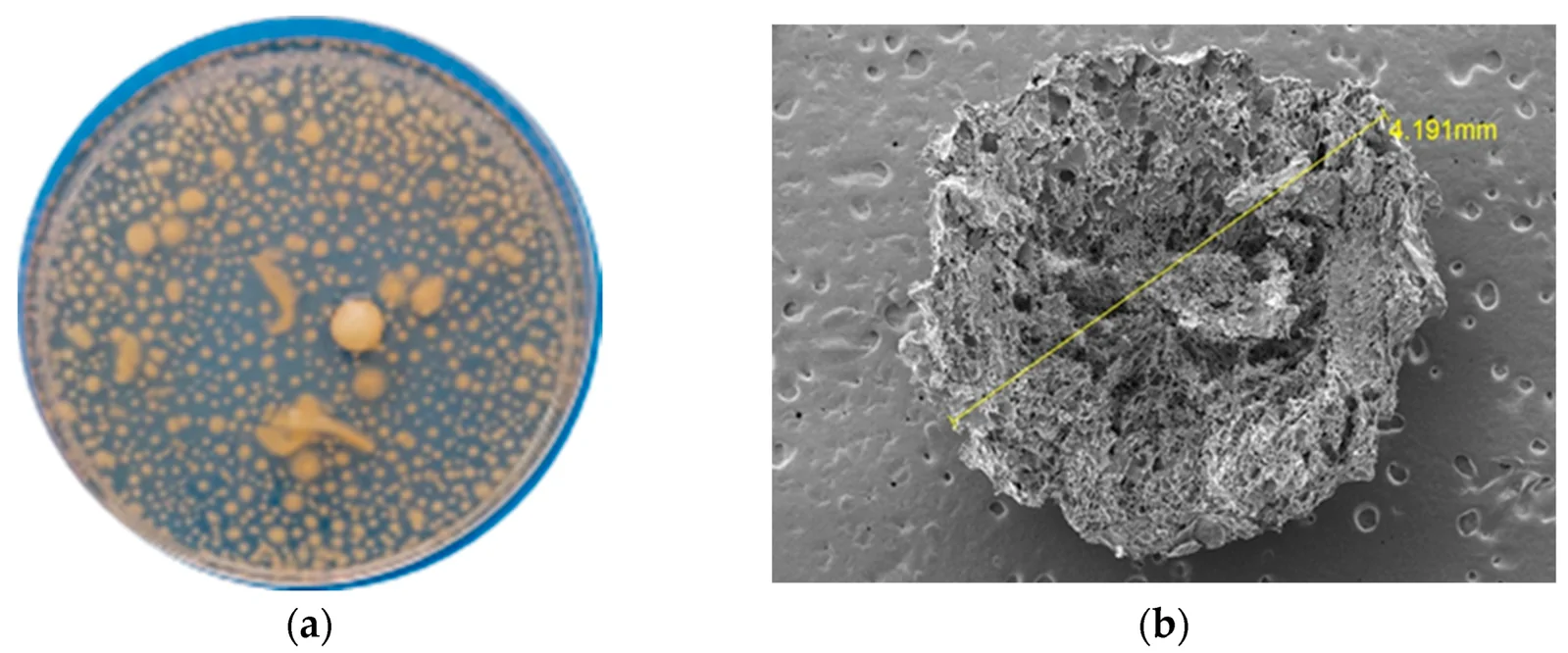
*P. salmoneostramineus* mycelium cultivated in WB4 under dynamic conditions, 200 rpm, 5 days: (**a**) photo of Petri dish 1×; (**b**) scanning electron microscopy 20×.

**Figure 4:**
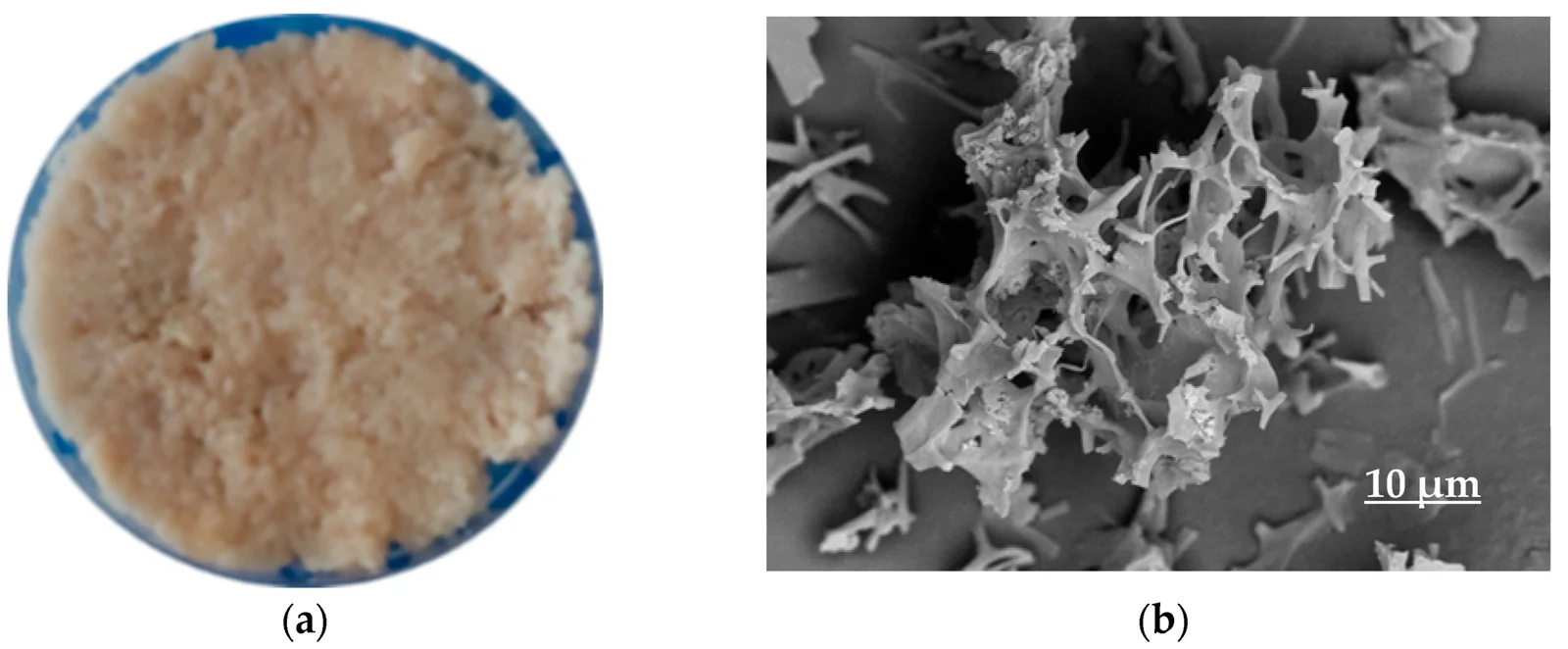
*P. salmoneostramineus* mycelium cultivated in WB4 + MBH5 under dynamic conditions, 200 rpm, 5 days: (**a**) photo of Petri dish 1×; (**b**) scanning electron microscopy 1000×.

The morphology of *P. salmoneostramineus* mycelium under different cultivation conditions is illustrated in Figure 1, Figure 2, Figure 3 and Figure 4. Under control static cultivation in PDB (Figure 1), the mycelium exhibited a loose filamentous network with widely distributed hyphae and open pore structures at 1000× magnification, characteristic of undisturbed growth in non-agitated systems. In contrast, mycelium cultivated in WB4 under static conditions (Figure 2) showed a more compact arrangement of ribbon-like hyphae, forming a denser filamentous network than in PDB. This may reflect the influence of substrate composition on hyphal organization. Under dynamic cultivation in WB4 (Figure 3), the mycelium formed spherical pellets with an average diameter of approximately 4.19 mm (observed at 20× magnification). Pellet formation is a common response in submerged fungal cultivation, as shear forces promote hyphal aggregation into dense spherical structures [29]. The rough and porous pellet surface (Figure 3b) suggests active hyphal growth at the periphery, where oxygen and nutrient availability are the highest.

In contrast, mycelium cultivated in WB4 + MBH5 under dynamic conditions (Figure 4) exhibited irregular clumps and flake-like morphologies, rather than well-defined pellets. At 1000× magnification, the structure appeared fragmented, with sheet-like formations and rough, irregular surfaces. This morphological transition may be due to the higher nutrient availability in the MBH-supplemented medium, which promoted rapid hyphal proliferation and growth, beyond that which could maintain structural stability of the pellets, resulting in fragmentation under continuous agitation [51]. The clump-like morphology observed under dynamic WB4 + MBH5 conditions provides a larger surface area for interaction with the surrounding medium, potentially enhancing nutrient uptake and contributing to the higher biomass (16.59 g/L) and protein content (66.71 g/100 g) observed under these conditions compared with WB4 alone.

Overall, the transition from dispersed mycelial networks under static conditions to pellet or clump structures under dynamic cultivation highlights the strong influence of process parameters on fungal morphology. Morphological characteristics, including pellet density and hyphal organization, directly affect mass transfer, downstream processing, and functional properties. Dense, interwoven hyphal structures may enhance water-holding capacity and fibrous texture, which are desirable attributes for mycoprotein-based food applications [1].

## 4. Conclusions

This study highlights the potential of *P. salmoneostramineus* in mycoprotein production using bread industry by-products under submerged cultivation conditions. A balanced C/N ratio (WB4; WBH:SBH 70:30; C/N 27.32:1) supported efficient growth under static conditions, while dynamic cultivation markedly enhanced productivity. WB4 supplemented with MBH (5 g/L) under dynamic cultivation (200 rpm, 5 days) achieved 16.59 g/L biomass with 66.71 g/100 g protein, substantially higher than values obtained under static cultivation (biomass 3.46 g/L, 14 days). This formulation also yielded high total amino acid (762.30 mg/g protein) and EAA contents (354.72 mg/g protein), meeting or exceeding FAO reference patterns. In addition, the relatively short cultivation period and the use of agro-industrial by-products highlight the potential of this system as a resource-efficient approach for sustainable protein production. Therefore, *P. salmoneostramineus*-based mycoprotein may serve as a promising complementary protein source in the emerging alternative protein market, supporting future food systems in response to increasing global protein demand. Future development should focus on process scale-up, especially regarding potential ribonucleic acid (RNA) accumulation in high-growth or continuous fermentation systems, which may pose food safety concerns due to its conversion to uric acid in humans. Although the batch fermentation applied in this study is less likely to promote excessive RNA accumulation, down-stream RNA reduction strategies may be required to meet the WHO-recommended threshold of ≤2% nucleic acid for food applications. Further studies should also evaluate functional properties to support the development of food products.

## Figures and Tables

**Table 1:** Total nitrogen, total sugar, and total amino acid composition of substrates.

Parameter	Substrate		
	WBH	SBH	MBH
Total Nitrogen (g/L)	2.58 ± 0.19 ^b^	1.32 ± 0.04 ^c^	3.58 ± 0.04 ^a^
Total Sugar (g/L)	6.44 ± 0.13 ^c^	57.53 ± 1.34 ^a^	22.89 ± 0.49 ^b^
Total Amino Acid (mg/100 mL)			
Acidic			
Aspartic acid	35.92	7.72	72.88
Glutamic acid	136.37	29.32	113.70
Basic			
Arginine	18.50	3.98	19.80
Histidine	10.08	2.17	16.01
Lysine	14.99	3.22	57.69
Hydrophobic			
Alanine	19.89	4.28	35.73
Glycine	25.80	5.55	38.23
Isoleucine	15.49	3.33	24.04
Leucine	26.99	5.80	40.99
Methionine	0.01	0.01	6.44
Proline	47.88	10.29	42.86
Cysteine	0.01	0.00	2.87
Valine	24.12	5.19	23.76
Aromatic			
Phenylalanine	21.83	4.69	18.91
Tyrosine	8.94	1.92	21.01
Hydrophilic			
Serine	24.66	5.30	39.29
Threonine	18.01	3.87	13.85
Total AA	449.47	96.64	588.06
Total EAA	150.02	32.25	215.05
Total HAA	160.17	34.44	205.61
Total EAA/% total AA ^a^	33.37	33.37	36.57
Total HAA/% total AA ^b^	35.63	35.64	34.96

Values of total nitrogen and total sugar are expressed as mean ± standard deviation (*n* = 3). Different superscript letters within the same row indicate significant differences (*p* < 0.05). WBH = wheat bran hydrolysate; SBH = stale bread hydrolysate; MBH = mung bean hydrolysate. ^a^ Total essential amino acid (EAA) values were calculated from the sum of arginine + lysine + histidine + threonine + valine + leucine + isoleucine + phenylalanine. ^b^ Total hydrophobic amino acids (HAA) were calculated from the sum of alanine + glycine + isoleucine + leucine + proline + valine.

**Table 2:** Effect of carbon source composition under static conditions on pH, residual sugar consumption, mycelium, and protein content at day 14.

Substrates (WBH:SBH)	C/N	pH	Residual Sugar Consumption (g/L)	Mycelium (g/L)	Protein Content (g/100 g)	Protein Production (g/L)
WB0 (PDB)	10:1	6.62 ± 0.20 ^a^	13.12 ± 0.04 ^g^	2.21 ± 0.09 ^c^	21.79 ± 0.58 ^b^	0.48 ± 0.01 ^c^
WB1 (100:0)	25.58:1	6.53 ± 0.18 ^b^	21.48 ± 0.09 ^d^	2.26 ± 0.21 ^c^	22.30 ± 0.40 ^b^	0.50 ± 0.01 ^c^
WB2 (90:10)	26.21:1	6.53 ± 0.17 ^b^	22.86 ± 0.13 ^b^	2.14 ± 0.42 ^c^	21.80 ± 0.67 ^b^	0.47 ± 0.02 ^d^
WB3 (80:20)	26.79:1	6.44 ± 0.31 ^c^	23.77 ± 0.08 ^a^	2.19 ± 0.05 ^c^	19.17 ± 0.39 ^c^	0.42 ± 0.04 ^a^
WB4 (70:30)	27.32:1	6.46 ± 0.31 ^c^	22.87 ± 0.66 ^b^	3.46 ± 0.26 ^a^	23.19 ± 0.88 ^a^	0.80 ± 0.00 ^cd^
WB5 (60:40)	27.80:1	6.40 ± 0.25 ^c^	22.54 ± 0.26 ^bc^	3.16 ± 0.89 ^ab^	13.98 ± 0.60 ^d^	0.44 ± 0.00 ^d^
WB6 (50:50)	28.24:1	6.63 ± 0.13 ^a^	20.50 ± 0.36 ^e^	2.12 ± 0.11 ^c^	18.14 ± 0.68 ^c^	0.38 ± 0.01 ^e^
WB7 (40:60)	28.65:1	6.69 ± 0.12 ^a^	19.99 ± 0.12 ^ef^	2.21 ± 0.06 ^c^	14.80 ± 0.08 ^d^	0.33 ± 0.01 ^e^
WB8 (30:70)	29.03:1	6.74 ± 0.04 ^a^	20.37 ± 0.18 ^e^	2.32 ± 0.35 ^bc^	14.14 ± 0.37 ^d^	0.33 ± 0.02 ^e^
WB9 (20:80)	29.39:1	6.73 ± 0.02 ^a^	20.01 ± 0.18 ^cd^	2.45 ± 0.07 ^b^	14.05 ± 0.16 ^d^	0.34 ± 0.04 ^e^
WB10 (10:90)	29.72:1	6.66 ± 0.10 ^ab^	19.93 ± 0.31 ^ef^	2.36 ± 0.26 ^bc^	13.46 ± 0.52 ^d^	0.32 ± 0.01 ^e^
WB11 (0:100)	30.03:1	6.58 ± 0.13 ^b^	19.39 ± 0.02 ^f^	2.30 ± 0.08 ^bc^	12.11 ± 0.47 ^d^	0.28 ± 0.02 ^c^

Values are expressed as mean ± standard deviation (*n* = 3). Different superscript letters within the same column indicate significant differences (*p* < 0.05).

**Table 3:** Effect of nitrogen sources under dynamic conditions on pH, residual sugar consumption, mycelium, and protein content at day 5.

Substrates	Dosage (g/L)	pH	Residual Sugar Consumption (g/L)	Mycelium (g/L)	Protein Content (g/100 g)	Protein Production (g/L)
WB0 (PDB)	10:1	6.52 ± 0.11	22.87 ± 0.66	5.12 ± 0.36	23.54 ± 0.12	1.20 ± 0.02
WB4 + MET	1	4.85 ± 0.11 ^d^	17.50 ± 0.26 ^h^	1.56 ± 0.36 ^g^	32.03 ± 0.59 ^e^	0.51 ± 0.05 ^h^
	3	5.38 ± 0.15 ^a^	21.31 ± 0.25 ^f^	2.38 ± 0.32 ^f^	37.37 ± 0.15 ^d^	0.90 ± 0.09 ^gh^
	5	4.67 ± 0.43 ^de^	20.46 ± 0.14 ^g^	2.28 ± 0.08 ^f^	31.32 ± 0.41 ^e^	0.72 ± 0.01 ^h^
WB4 + GLY	1	5.37 ± 0.02 ^a^	18.44 ± 1.73 ^h^	5.27 ± 0.18 ^e^	30.32 ± 0.28 ^e^	1.59 ± 0.01 ^fg^
	3	5.25 ± 0.01 ^ab^	19.35 ± 0.21 ^g^	5.54 ± 0.25 ^d^	32.52 ± 0.26 ^e^	1.81 ± 0.04 ^f^
	5	5.26 ± 0.03 ^ab^	19.39 ± 0.26 ^g^	9.68 ± 0.30 ^d^	32.43 ± 0.57 ^e^	3.15 ± 0.02 ^e^
WB4 + PEP	1	4.52 ± 0.03 ^e^	19.63 ± 0.18 ^g^	7.65 ± 0.32 ^d^	57.21 ± 0.48 ^b^	4.37 ± 0.06 ^de^
	3	4.67 ± 0.08 ^de^	19.68 ± 0.32 ^gh^	9.43 ± 0.62 ^c^	53.52 ± 0.35 ^c^	5.06 ± 0.18 ^d^
	5	4.54 ± 0.12 ^e^	18.59 ± 0.20 ^e^	11.51 ± 0.73 ^cd^	66.93 ± 0.20 ^a^	7.73 ± 0.32 ^b^
WB4 + MBH	1	4.45 ± 0.04 ^e^	25.57 ± 0.22 ^e^	10.22 ± 0.51 ^cd^	47.22 ± 0.67 ^c^	4.83 ± 0.09 ^d^
	3	4.53 ± 0.05 ^e^	30.36 ± 0.30 ^c^	11.30 ± 0.54 ^c^	61.32 ± 0.90 ^ab^	6.99 ± 0.31 ^c^
	5	4.71 ± 0.31 ^cde^	35.43 ± 0.36 ^a^	16.59 ± 0.26 ^a^	66.71 ± 0.86 ^a^	11.07 ± 0.09 ^a^

Values are mean ± standard deviation (*n* = 3). Different superscript letters within the same column indicate significant differences (*p* ≤ 0.05) according to Duncan’s multiple range test. MET = methionine; GLY = glycine; PEP = soy peptone; MBH = mung bean hydrolysate.

**Table 4:** Total amino acid composition (mg/g protein) of *P. salmoneostramineus* mycoprotein produced under static WB4, dynamic WB4, and dynamic WB4 + MBH5 conditions, compared with FAO/WHO reference amino acid scoring patterns.

Total Amino Acid (mg/g Protein)	Static WB4	Dynamic WB4	Dynamic WB4 + MBH5	Recommended Amino Acid Scoring (FAO) (mg/g Protein)	
				Child (6 Months to 3 years)	Older Child, Adolescent, and Adult
Acidic					
Aspartic acid	13.12	35.57	94.04	-	-
Glutamic acid	15.66	51.98	109.18	-	-
Basic					
Arginine	3.39	13.12	47.51	-	-
Histidine	4.23	8.66	17.94	-	-
Lysine	5.93	20.06	50.88	57	48
Hydrophobic					
Alanine	8.47	23.26	61.25	-	-
Glycine	6.77	20.06	43.03	-	-
Isoleucine	5.50	15.50	39.94	32	30
Leucine	8.47	26.45	67.41	8.5	6.6
Proline	10.58	23.26	53.68	-	-
Valine	6.77	20.06	43.03	43	40
Aromatic					
Phenylalanine	5.08	16.87	39.24	5.47	39.24
Tyrosine	2.54	7.30	27.05	2.74	27.05
Hydrophilic					
Serine	7.62	21.43	38.82		
Threonine	6.35	19.15	38.12		
Total AA	109.21	320.11	762.30		
Total EAA	49.53	143.17	354.72		
Total HAA	45.29	125.86	299.51		
Total EAA/% total AA ^a^	45.35	44.71	46.53		
Total HAA/% total AA ^b^	41.47	39.33	39.29	-	-

^a^ Total essential amino acid (EAA) values were calculated from the sum of arginine + lysine + histidine + threonine + valine + leucine + isoleucine + phenylalanine. ^b^ Total hydrophobic amino acids (HAA) were calculated from the sum of alanine + glycine + isoleucine + leucine + proline + valine.

## Data Availability

The original contributions presented in this study are included in the article/Supplementary Material; further inquiries can be directed to the corresponding author.

## Supplementary Materials

The following supporting information can be downloaded at: https://www.mdpi.com/article/10.3390/foods15101773/s1, Table S1: Total amino acid composition of raw materials (WB, SB, and MBPI). Table S2. Effect of carbon source formulations on pH and biomass of *P. salmoneostramineus* under static cultivation at day 7. Table S3. Total amino acid composition of WB4 medium and WB4 + MBH5 medium.
