# Supplementary material for: Valorization of Bread Waste Hydrolysates and Plant-Based Nitrogen Sources for Mycoprotein Production by Pleurotus salmoneostramineus
Source: Foods. 2026 May 17;15(10):1773. doi: 10.3390/foods15101773 (PMC13206093; doi:10.3390/foods15101773)
Supplement: Supplementary file 1 [file foods-15-01773-s001.zip › foods-4284019-supplementary.pdf]

Table S1. Total amino acid composition of raw materials (WB, SB, and MBPI).

Amino Acids		WB (mg/100mg)	SB (mg/100mg)	MBPI (mg/100mg)
**Acidic (- charge)**	Aspartic acid	0.98	0.52	2.41
	Glutamic acid	2.92	4.28	3.97
**Basic (+charge)**	Arginine	0.88	0.38	0.43
	Histidine	0.38	0.27	0.52
	Lysine	0.57	0.19	1.26
**Hydrophobic**	Alanine	0.69	0.42	0.80
	Glycine	0.84	0.51	0.70
	Isoleucine	0.50	0.50	0.57
	Leucine	0.98	0.96	1.34
	Methionine	ND	ND	0.91
	Proline	1.08	1.69	0.22
	Cysteine	ND	ND	0.73
	Valine	0.84	0.65	0.14
**Aromatic**	Phenylalanine	0.68	0.74	1.19
	Tyrosine	0.36	0.31	0.73
**Hydrophilic**	Serine	0.75	0.71	0.96
	Threonine	0.53	0.41	0.59
	Total AA	12.98	12.54	17.46
	Total EAA	5.36	4.1	6.04
	Total HAA	4.93	4.73	3.70
	Total EAA/% total AA[a]	41.29	32.69	34.57
	Total HAA/% total AA[b]	37.98	37.71	21.58

a Total essential amino acid (EAA) values were calculated from the sum of arginine + lysine + histidine + threonine + valine + leucine + isoleucine + phenylalanine.

b Total hydrophobic amino acids (HAA) were calculated from the sum of alanine + glycine + isoleucine + leucine + proline + valine.

WB = Wheat bran; SB = Stale bread; MBPI = Mung bean protein isolate. ND =Not Detected

Table S2. Effect of carbon source formulations on pH and biomass of *P. salmoneostramineus* under static cultivation at day 7.

Substrate	C/N	pH	Biomass (g/L)
		Day 7	
**WB0 (PDB)**	10:1	5.27 ± 0.22^{c}	1.60 ± 0.50^{b}
**WB1 (100:0)**	25.58:1	5.92 ± 0.09^{a}	1.12 ± 0.09^{c}
**WB2 (90:10)**	26.21:1	5.75 ± 0.21^{a}	0.65 ± 0.29^{d}
**WB3 (80:20)**	26.79:1	5.47 ± 0.45^{b}	1.07 ± 0.15^{c}
**WB4 (70:30)**	27.32:1	5.78 ± 0.11^{a}	2.30 ± 0.28^{a}
**WB5 (60:40)**	27.80:1	4.50 ± 0.36^{e}	1.43 ± 0.46^{b}
**WB6 (50:50)**	28.24:1	4.36 ± 0.26^{e}	1.68 ± 0.24^{b}
**WB7 (40:60)**	28.65:1	5.71 ± 0.24^{a}	1.34 ± 0.29^{bc}
**WB8 (30:70)**	29.03:1	4.54 ± 0.20^{e}	1.46 ± 0.73^{b}
**WB9 (20:80)**	29.39:1	4.51 ± 0.37^{e}	1.32 ± 0.48^{bc}
**WB10 (10:90)**	29.72:1	4.73 ± 0.14^{de}	0.95 ± 0.49^{cd}
**WB11 (0:100)**	30.03:1	4.90 ± 0.02^{d}	0.76 ± 0.25^{d}

Values are expressed as mean ± standard deviation (n = 3). Different superscript letters within the same column indicate significant differences at $p \leq 0.05$.

Table S3. Total amino acid composition of WB4 medium and WB4+MBH5 medium.

**Amino Acids**		**WB4 (mg/100mL)**	**WB4+MBH (mg/100mL)**
**Acidic (- charge)**	Aspartic acid	44.9	159.95
	Glutamic acid	170.46	351.01
**Basic (+charge)**	Arginine	23.13	49.5
	Histidine	12.6	31.32
	Lysine	18.74	83.97
**Hydrophobic**	Alanine	24.86	71.04
	Glycine	32.25	74.5
	Isoleucine	19.36	52.5
	Leucine	33.74	94.53
	Methionine	0.01	0.00
	Proline	59.85	97.13
	Cysteine	0.01	0.00
	Valine	30.15	68.03
**Aromatic**	Phenylalanine	27.29	74.38
	Tyrosine	11.17	64.6
**Hydrophilic**	Serine	30.83	86.97
	Threonine	22.51	84.33
	Total AA	561.86	1443.76
	Total EAA	187.52	538.56
	Total HAA	200.21	457.73
	Total EAA/% total AA[a]	33.37	37.30
	Total HAA/% total AA[b]	35.63	31.70

a Total essential amino acid (EAA) values were calculated from the sum of arginine + lysine + histidine + threonine + valine + leucine + isoleucine + phenylalanine.
b Total hydrophobic amino acids (HAA) were calculated from the sum of alanine + glycine + isoleucine + leucine + proline + valine.
WB4 medium = WBH:SBH (70:30); WB4+MBH5 = WBH:SBH (70:30) supplemented with mung bean hydrolysate at 5 g/L.
